# Formulation matters: LION nanoparticles improve RNA vaccine immunogenicity by containing systemic inflammation

**DOI:** 10.1016/j.omtn.2025.102659

**Published:** 2025-08-11

**Authors:** Chandru Subramani, Tarani Kanta Barman

**Affiliations:** 1 Department of Pathology, University of Texas Medical Branch, Galveston, TX, USA; 2 Galveston National Laboratory, Galveston, TX, USA

## Main text

The success of SARS-CoV-2 mRNA vaccines during the pandemic is driving the evolution of potential next-generation mRNA platforms, such as circular RNA and replicon RNA (repRNA), to prolong antigen expression, which could augment the immune response even at lower doses than conventional mRNA vaccines.^1^^,^^2^^,^^3^^,^^4^ While lipid nanoparticles (LNPs) are the most commonly used nanoformulation for delivering mRNA, they possess side effects. In contrast, the lipid-inorganic nanoparticle (LION) is emerging as an alternative to mitigate LNP-associated severe inflammation and enhance tolerability.^5^ Recently, improved LNPs containing anti-inflammatory endogenous lipid nitro-oleic acid have been reported to ameliorate acute inflammation and prolong transgene expression in a mouse model.^6^ In this context, a recent study published in *Molecular Therapy – Nucleic Acids* by Kimura et al. investigated how a repRNA vaccine with either LNP or LION formulations affects the systemic immune response, showing a negative correlation between early systemic interferon (IFN) levels and both binding and neutralizing antibody titers.^7^

The authors employed a multicomponent repRNA vaccine encoding virus-like particles representing three genotypes of enterovirus D68 (EV-D68) (subclade A1, B1, and C) and the F and G glycoproteins of respiratory syncytial virus (RSV). repRNA vaccines are self-amplifying repRNA that encodes its replication machinery, as well as the vaccine antigen, enabling even lower doses to induce a potent and sustained immune response than conventional non-replicating mRNA vaccines. The authors designed the study based on their prior publication, which showed in mice that repRNA formulated with LNP (repRNA/LNP) was distributed across multiple organs, inducing systemic innate responses, including excessive type I IFN response.^8^ In contrast, repRNA formulated with LION (repRNA/LION) was localized in the muscles and draining lymph nodes, inducing a robust local innate response. The present study extends those findings to a more clinically relevant pigtail macaque model.

In macaques, the repRNA/LNP formulation induced a rapid systemic IFN-α2 response and upregulated genes associated with chemokines and IFN pathways in peripheral blood mononuclear cells at day 1 post-vaccination. The pathway analysis further revealed enrichment of IFN signaling, Toll-like receptors, and innate immune activation. In contrast, the repRNA/LION formulation exhibited significantly lower levels of IFN-α2, and only a few genes were upregulated, which are involved in adaptive immunity. Despite comparable antibody responses elicited to the RSV-F and RSV-G antigens in both formulations, only repRNA/LION elicited binding IgG and neutralizing antibody responses against EV-D68. Furthermore, the study found a significant negative correlation between serum IFN-α2 levels and the EV-D68 binding IgG response. The authors supported this observation by analyzing their previous mouse study, as well as macaque data, which both showed a subclade-specific (B1 and C) negative correlation between serum IFN-α2 levels and neutralizing antibody response. Overall, the study suggests that a systemic inflammatory immune response may negatively affect the humoral response against specific antigens, such as EV-D68, as the RSV antigens retained their immunogenicity regardless of formulation. LION’s ability to localize immune activation while maintaining high tolerability may enable the safe and effective delivery of high-dose, multicomponent repRNA vaccines. Although the study offers important insights, it does not include tissue-specific inflammatory markers or T cell responses, nor does it examine the involvement of B cells, macrophages, and dendritic cells at the site of injection.

In conclusion, the study primarily focused on how nanoformulation impacts local versus systemic inflammatory responses, which in turn affects humoral antibody responses to vaccine antigens. Future studies may investigate how these differential inflammatory responses affect CD4^+^ and CD8^+^ T cell responses, as cellular immunity plays a crucial role in protecting against viruses. Additionally, challenge studies will reveal how LNP and LION formulations influence protective efficacy *in vivo*. Lastly, the mechanistic insights for antigen-specific and subclade-specific differences in antibody responses remain speculative and need further investigation.

## Declaration of interests

The authors declare no competing interests.
